# Supplementary figures and images for: Intermittent bulk release of human cytomegalovirus
Source: PLoS Pathog. 2022 Aug 4;18(8):e1010575. doi: 10.1371/journal.ppat.1010575 (PMC9352052; doi:10.1371/journal.ppat.1010575)

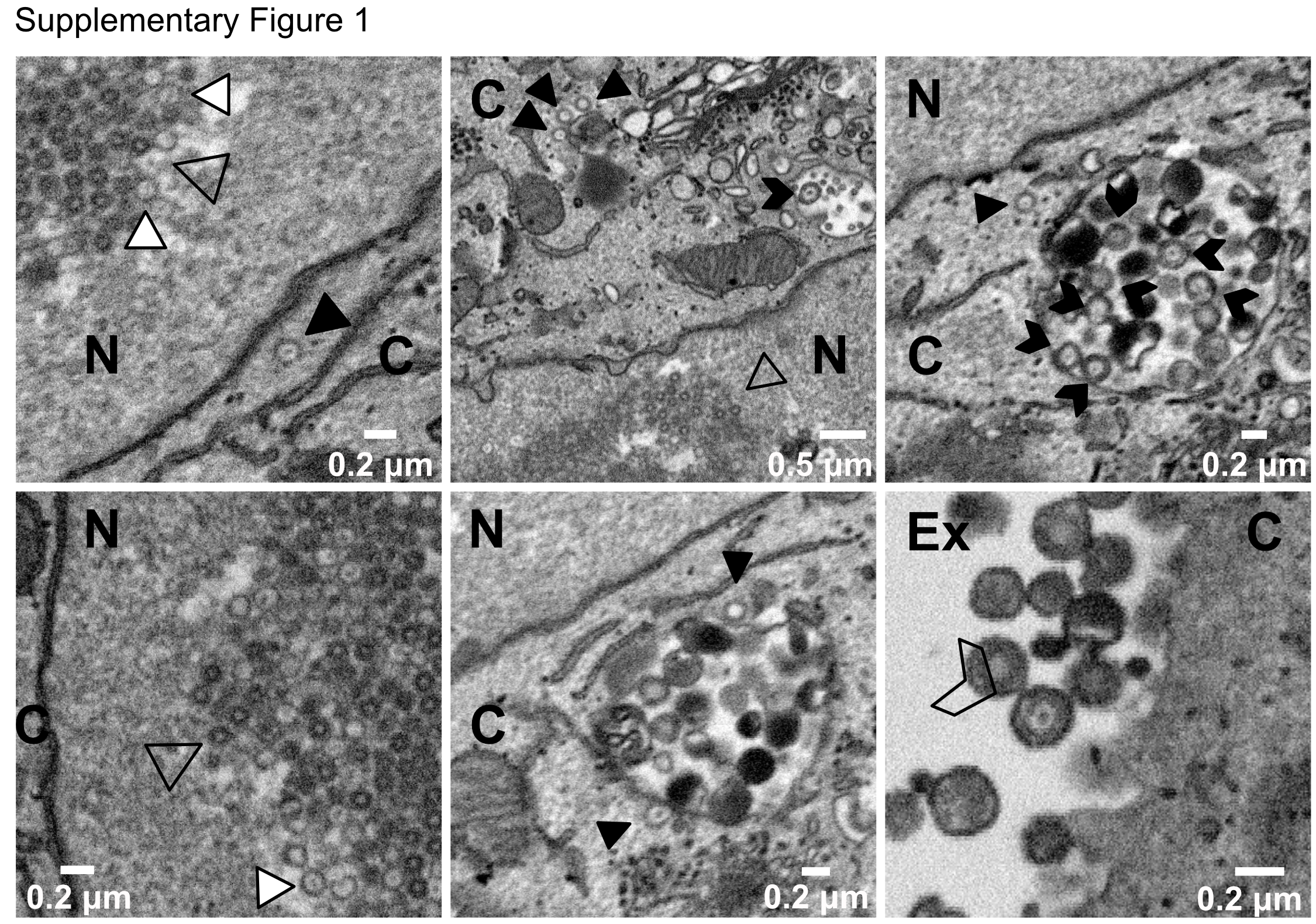

Supplement: S1 Fig — HFF cells were infected with HCMV-pp150-EGFP-gM-mCherry (MOI 3) and processed for EM 4 dpi. Image signals were inverted to facilitate comparison with TEM images. N marks nucleoplasm, C indicates cytoplasm, and Ex the extracellular space. Highlighted in the panels are examples of B-capsids in the nucleus (unfilled black triangles), DNA- filled nuclear C-capsids (white triangles with black contour), cytoplasmic non-enveloped C-capsids (black triangles), intracellular, enveloped virus particles (black filled arrowheads) as well as enveloped, released particles (empty arrowhead with black contour). Scale bar lengths are specified in each image. (TIF) [file ppat.1010575.s001.tif]

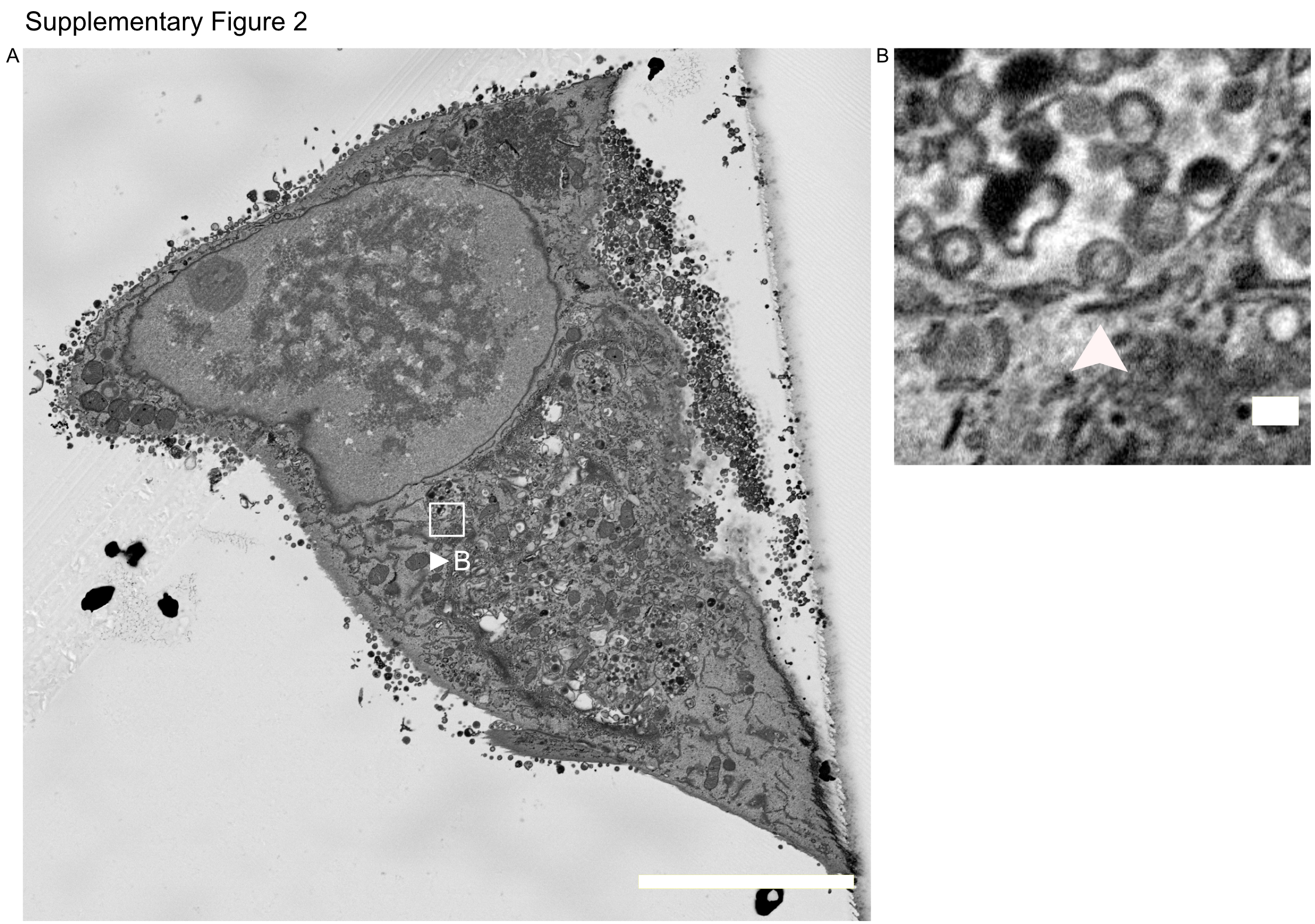

Supplement: S2 Fig — S2A Single SBF-SEM section of an infected HFF cell. HFF cell infected with HCMV-pp150-EGFP-gM-mCherry (MOI 3) at 4dpi. Image signals were inverted to facilitate comparison with TEM images. The white frame indicates the area cropped and enlarged in B, showing the surface of an MViB in the periphery of the assembly complex. Scale bar indicates 10 μm. S2B A detail showing a single particle potentially budding into an MViB (white arrowhead). Scale bar indicates 200nm. (TIF) [file ppat.1010575.s002.tif]

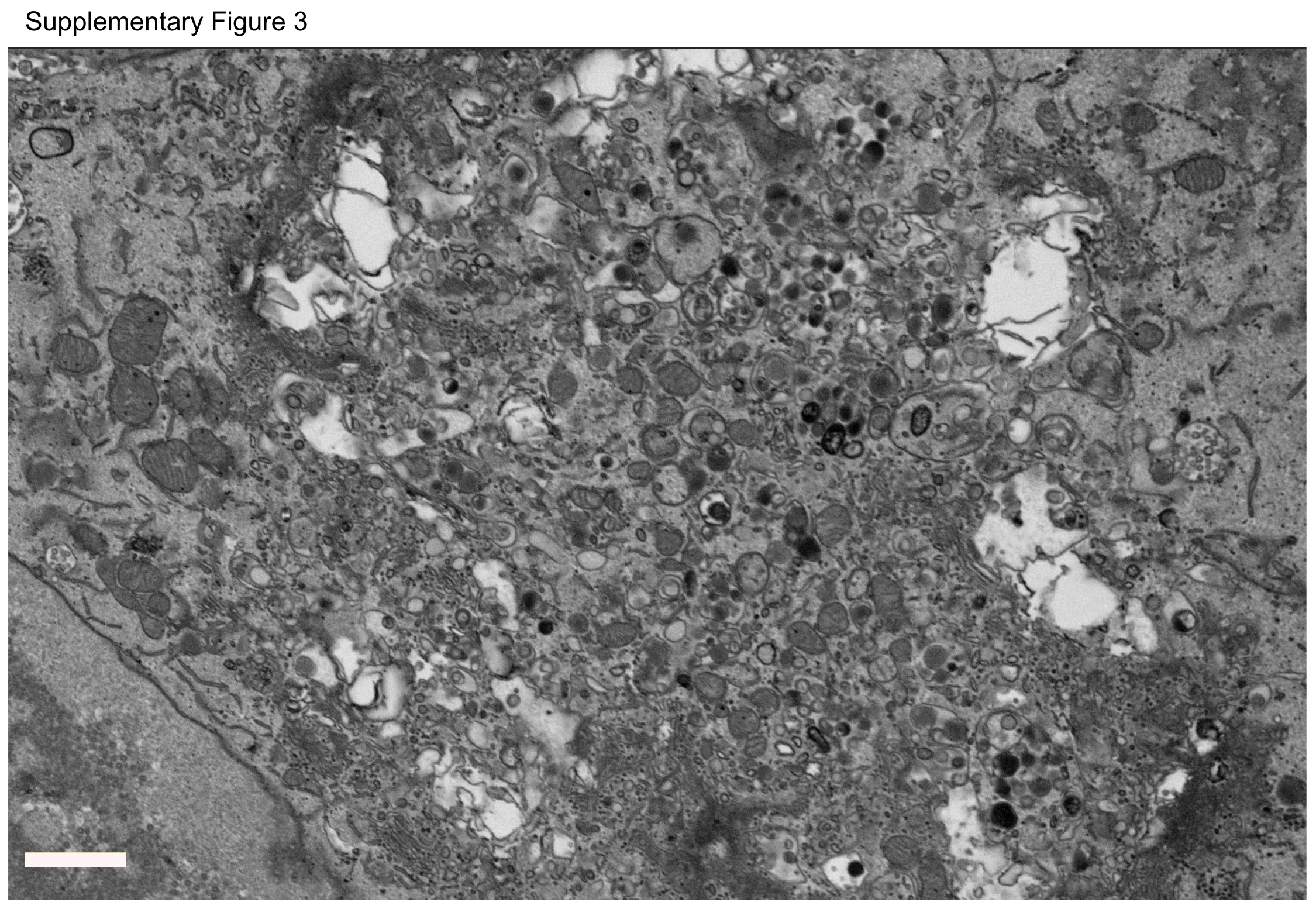

Supplement: S3 Fig — HFF cell infected with HCMV-pp150-EGFP-gM-mCherry (MOI 3) at 4dpi. Shown is the assembly complex in a resliced section through an SBF-SEM stack. Scale bar indicates 1.5 μm. Image signals were inverted to facilitate comparison with TEM images. (TIF) [file ppat.1010575.s003.tif]

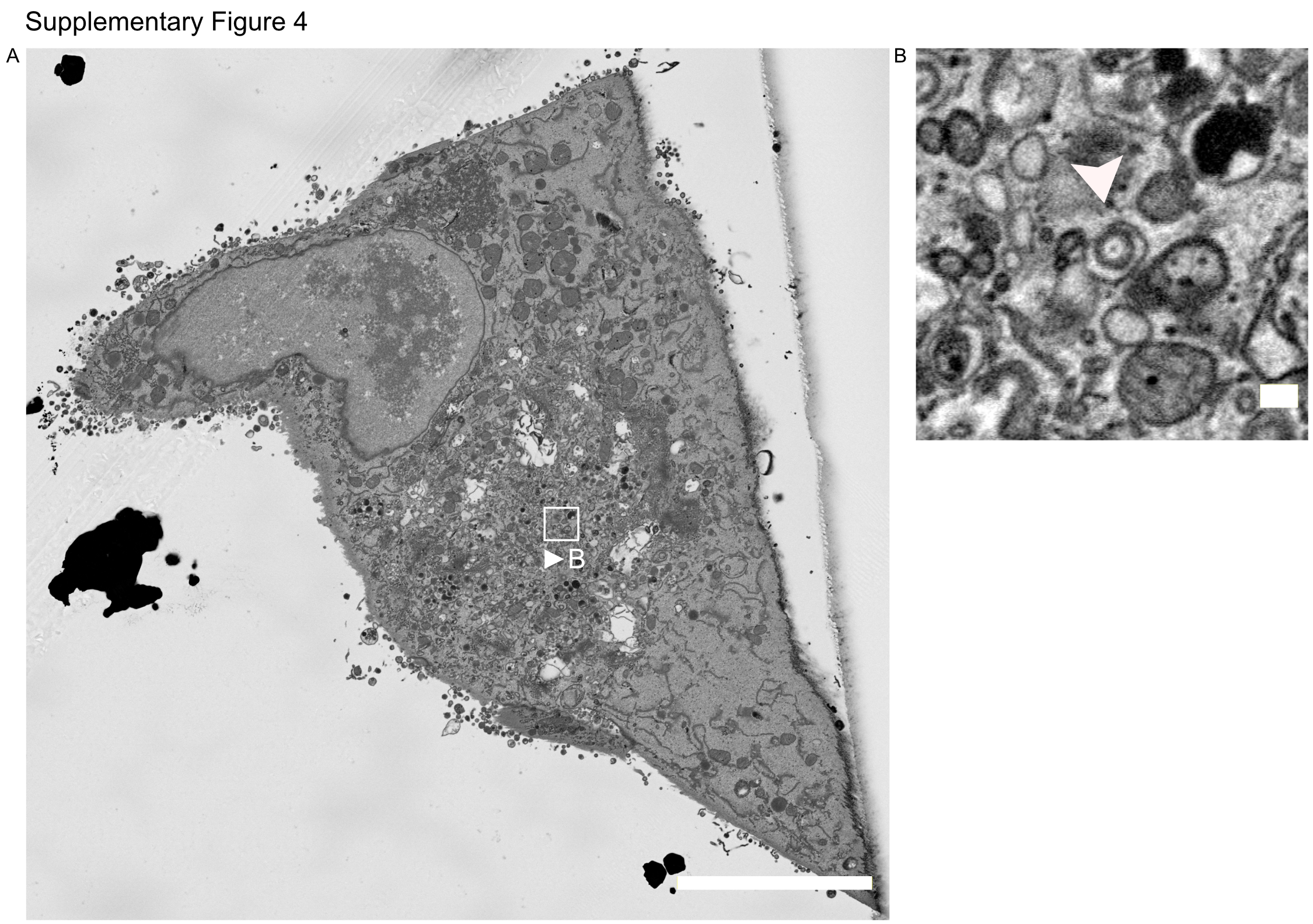

Supplement: S4 Fig — S4A Single SBF-SEM section of an infected HFF cell. HFF cell infected with HCMV-pp150-EGFP-gM-mCherry (MOI 3) at 4dpi. The white frame indicates the area cropped and enlarged in B. Scale bar indicates 10 μm. S4B A detail showing a single capsid budding into a single vesicle (white arrowhead). Scale bar indicates 200 nm. Image signals were inverted to facilitate comparison with TEM images. (TIF) [file ppat.1010575.s004.tif]

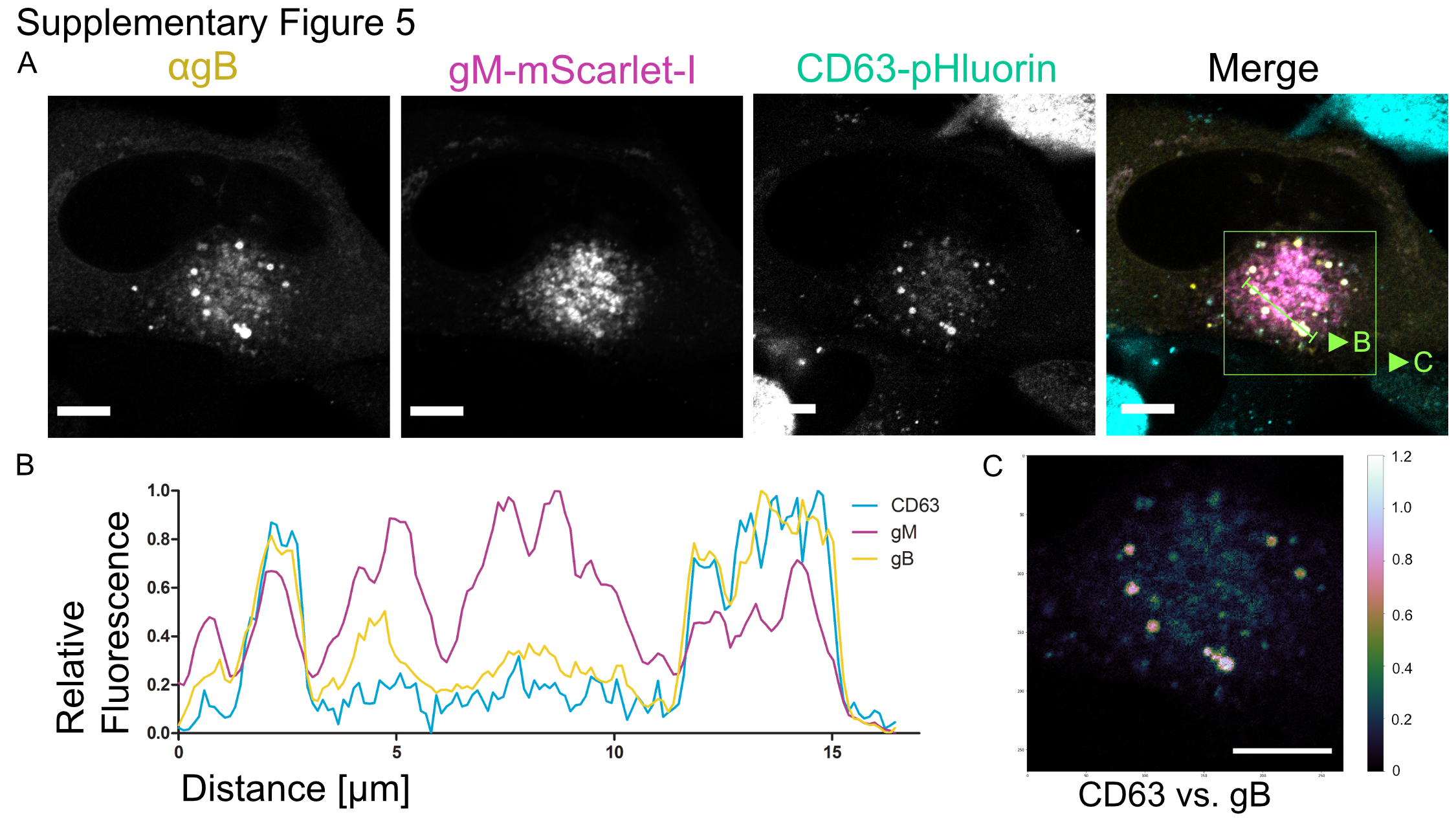

Supplement: S5 Fig — S5A HFF-CD63-pHluorin cells were infected at an MOI of 1 with HCMV-pp150-SNAP-gM-mScarlet-I. Cells were fixed at 4 dpi and stained for gB. The images show a representative cell and the localization pattern of the cellular MVB marker CD63 in relation to the viral glycoproteins gB and gM. CD63 localizes to large vesicles positive for gB and gM. Scale bars indicate 10 μm. The green line indicates the section quantified in S5B. S5B Line plot for the indicated areas in S5A. CD63 signal correlates with gM and gB signals in two MViBs. S5C Spatial weighted colocalization analysis highlights the specific areas for CD63 and gB colocalization. Scale bar indicates 10 μm. (TIF) [file ppat.1010575.s005.tif]

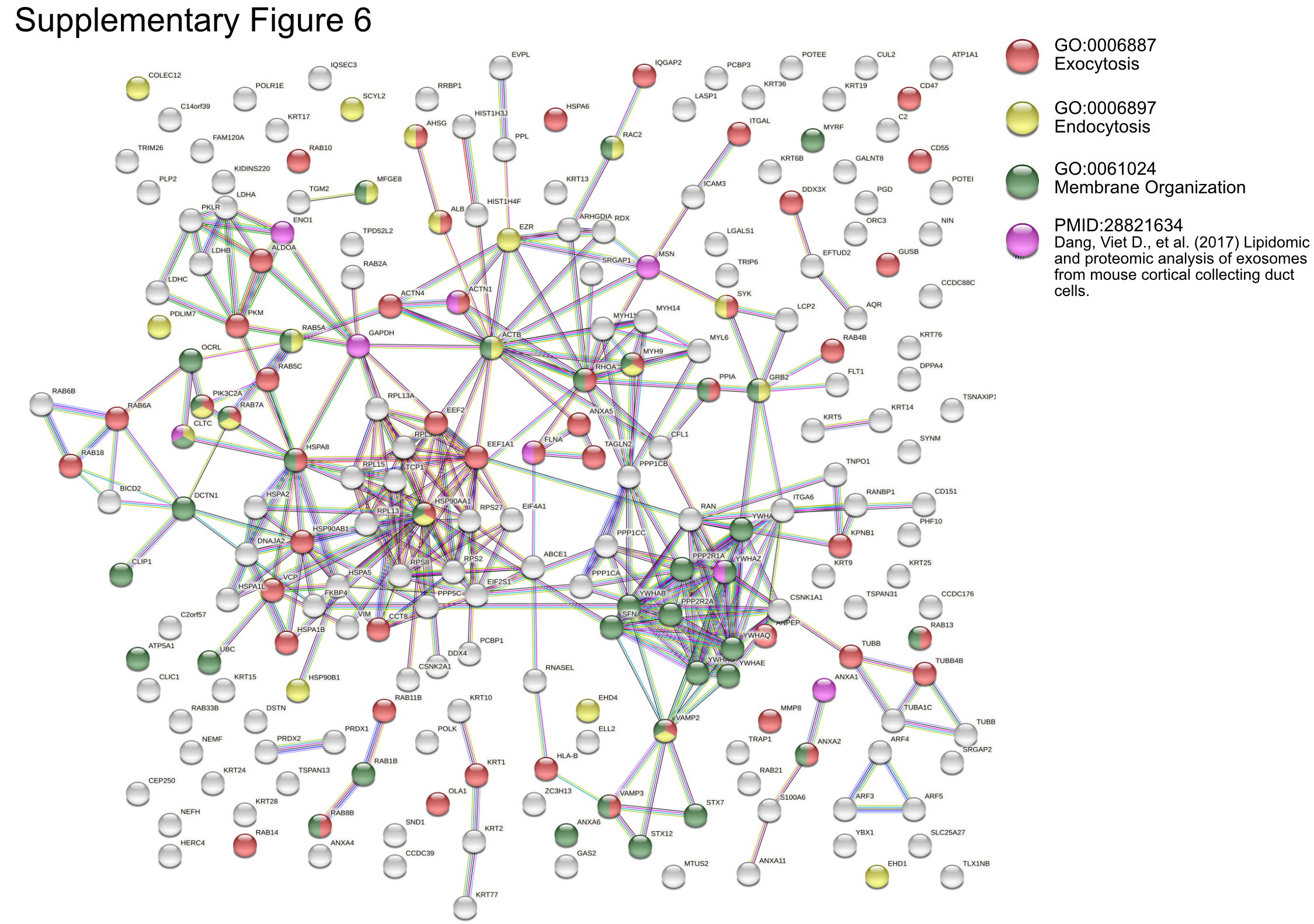

Supplement: S6 Fig — Pathway analysis of the mass spectrometry data from purified virions, done with string-db.org. The color of the dots indicates factors from either GO-term associated pathways or publications related to their functionality. The colors of the connections indicate the type of evidence for the interactions and are filtered for the highest interaction confidence (0.900) as provided by the database. (TIF) [file ppat.1010575.s006.tif]

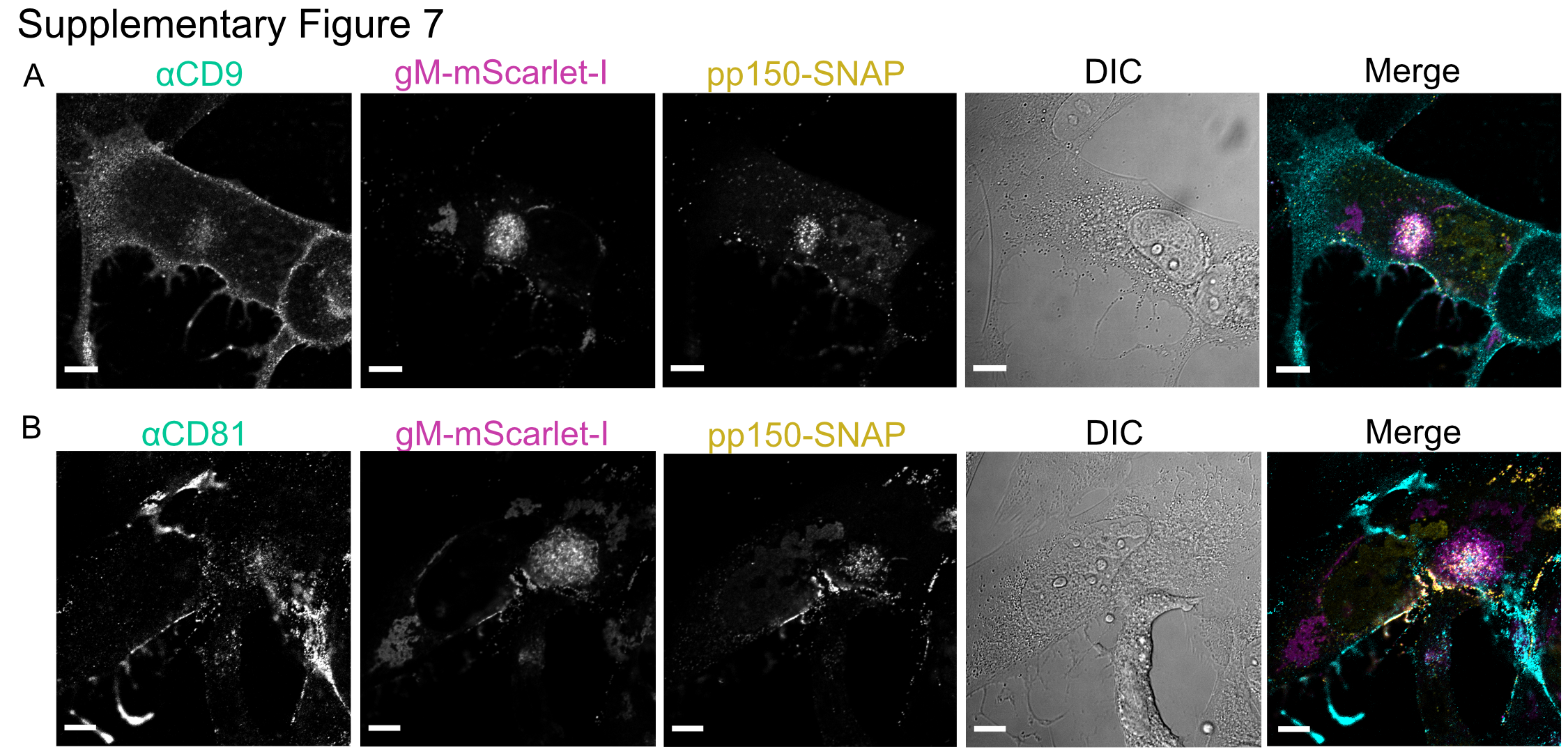

Supplement: S7 Fig — S7A-B HFF cells were infected at an MOI of 1 with HCMV-pp150-SNAP-gM-mScarlet-I. Cells were fixed at 4 dpi and stained with specific antibodies for CD9 (⍺CD9) and CD81 (⍺CD81). The images show representative cells and the localization pattern of the CD molecules relative to gM (gM-mScarlet-I) and pp150 (pp150-SNAP). Scale bars indicate 10 μm. (TIF) [file ppat.1010575.s007.tif]

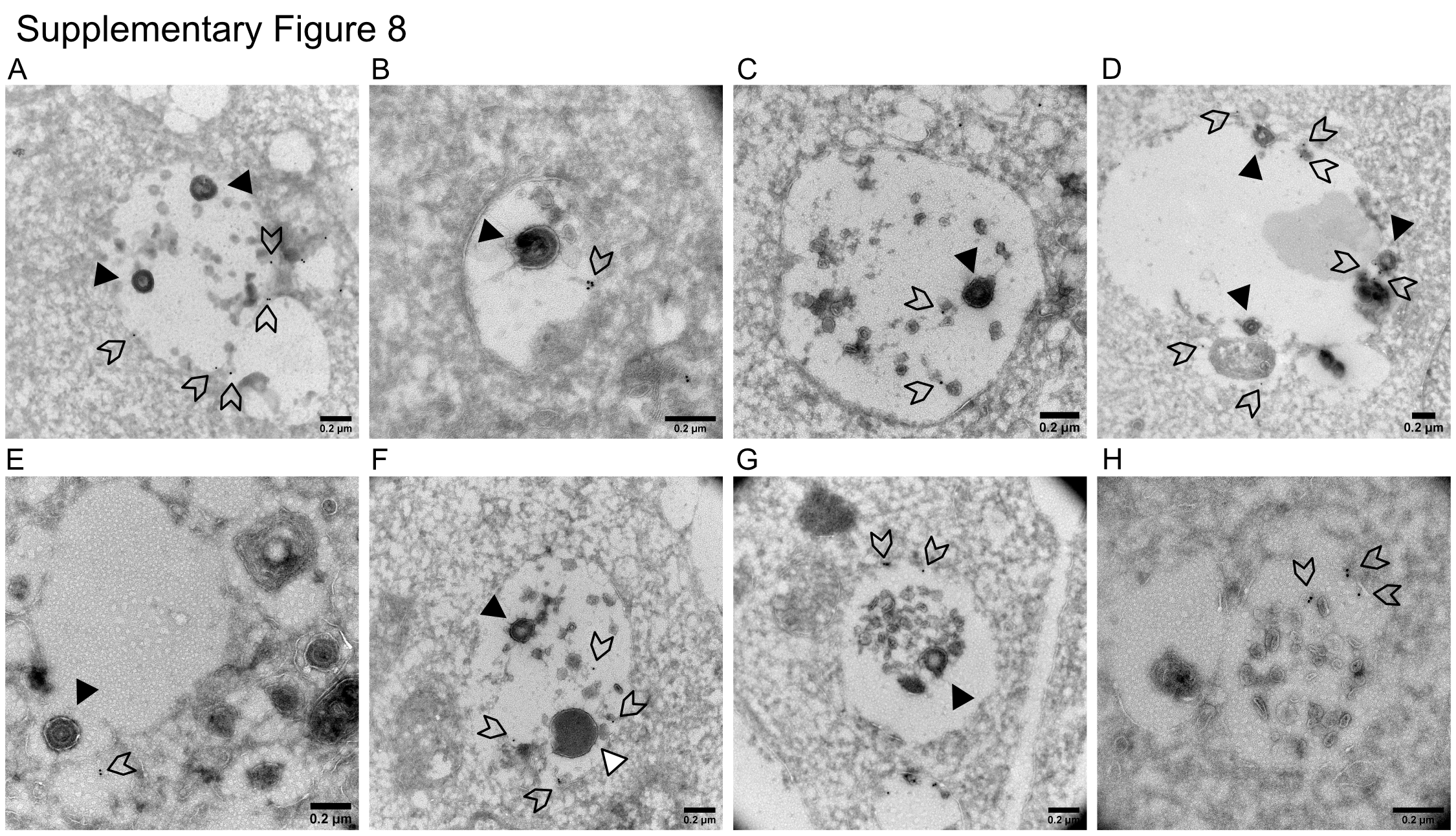

Supplement: S8 Fig — HFF cells were infected with HCMV-TB40-WT at an MOI of 0.5. After 4 dpi, the cells were fixed and processed for immunogold labeling against CD63. Note that membranes appear white in this preparation method, and low GA concentrations used to preserve epitopes might lead to less preservation of MViB contents. S8A-G Shown is large bodies containing virus particles (black triangles), dense bodies (white-filled triangles), and 10 nm gold particles (arrowheads). S8H A body with the classical phenotype of an MVB decorated with 10 nm gold particles (arrowheads). All scale bars indicate 0.2 μm. (TIF) [file ppat.1010575.s008.tif]

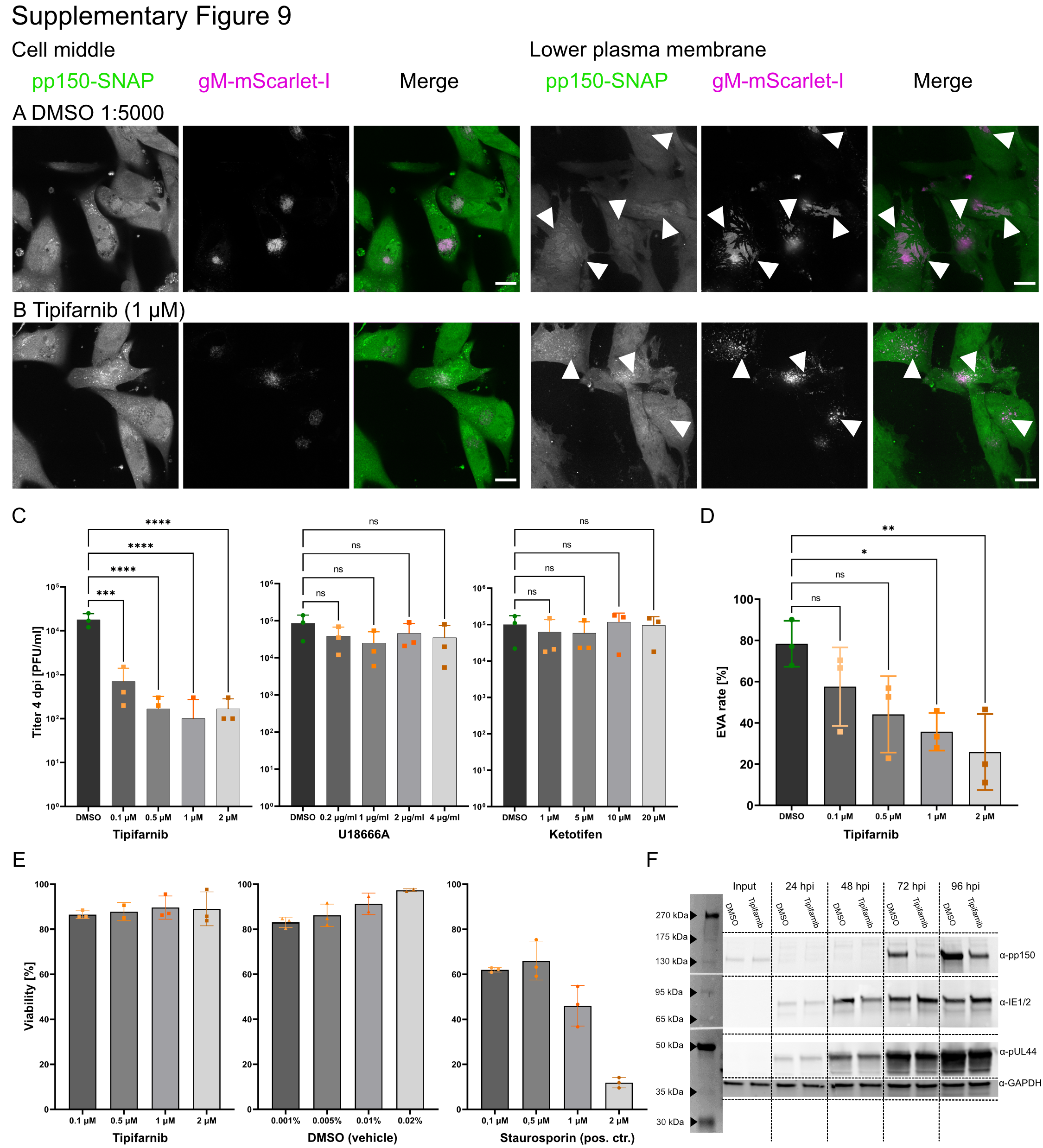

Supplement: S9 Fig — The medium containing the inhibitors was refreshed every 24 hours. 5 dpi cells were fixed, labeled with SNAP-Cell-SiR, and imaged by spinning-disk microscopy. White triangles indicate EVAs. Scale bars indicate 20 μm. S9C HFF cells were infected with HCMV-TB40-pp150-SNAP-gM-mScarlet-I at an MOI of 2 and treated with the indicated inhibitors at the indicated concentrations until 4 dpi. The medium containing the inhibitors was refreshed every 24 hours. At 4 dpi, the supernatant was collected and titrated on HFF cells. Bars show mean, and error bars indicate standard deviation. Statistical significance was probed using one-way ANOVA (p-values: Tipifarnib: <0.0001, U18666A: 0.4154, Ketotifen: 0.8364) and Dunnet’s multiple comparisons tests (shown in the figure). S9D HFF cells were treated as described for S9 A-B. Large overviews were created by spinning-disk microscopy, and EVAs were quantified. Bars show mean, and error bars indicate standard deviation. Statistical significance was calculated using a one-way ANOVA (p = 0.0179; in total, 687 late infected cells from triplicates were counted) and Dunnet’s multiple comparisons test (shown in the figure). S9E HFF were treated with the indicated substance at the indicated concentration for 24 hours. Cell viability was measured with an ATP assay after 24 h. The apoptosis inducer Staurosporine was used as a positive control. Bars show mean, and error bars indicate standard deviation. Statistical analysis by a 2-way ANOVA confirmed statistically significant differences in the viabilities of the three groups (p-value < 0.0001). The cytotoxicity of Tipifarnib was not significantly different from the vehicle control, as determined by Tukey’s multiple comparisons test. In contrast, the change in cell viability of Staurosporin was significant in the same analysis. S9F Western blot of HFF cells infected with HCMV-TB40-WT (MOI = 3) and treated with 1 μM Tipifarnib or DMSO (0.01%; vehicle control). At each indicated time point (input = [file ppat.1010575.s009.tif]

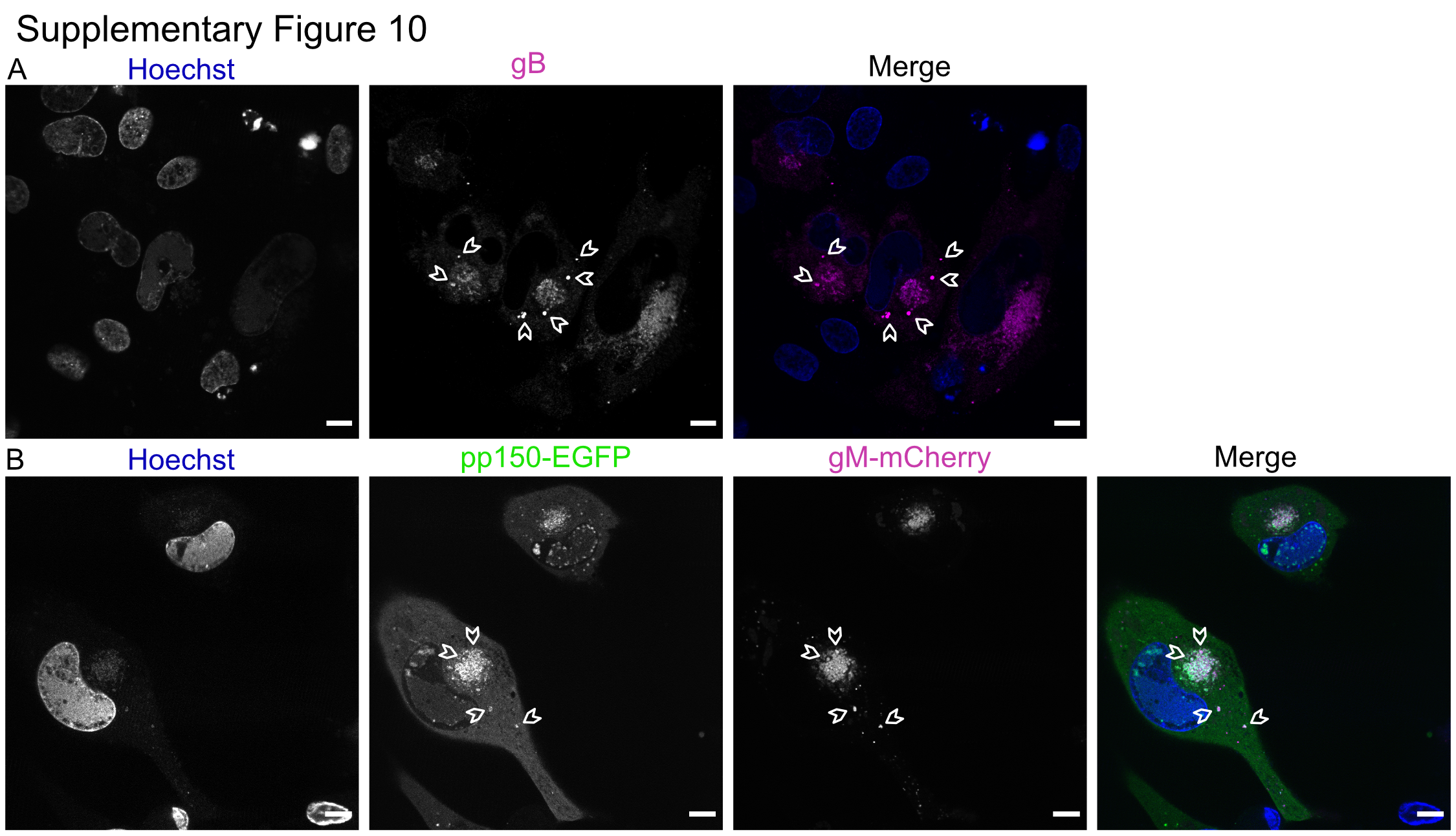

Supplement: S10 Fig — HFF-cells were infected as described in Fig 6B and 6C. Large cytoplasmic vesicles positive for viral proteins (white arrowheads) could be found in infection with both Merlin-WT (S10A) as well as Merlin-pp150-EGFP-gM-mCherry (S10B). (TIF) [file ppat.1010575.s010.tif]

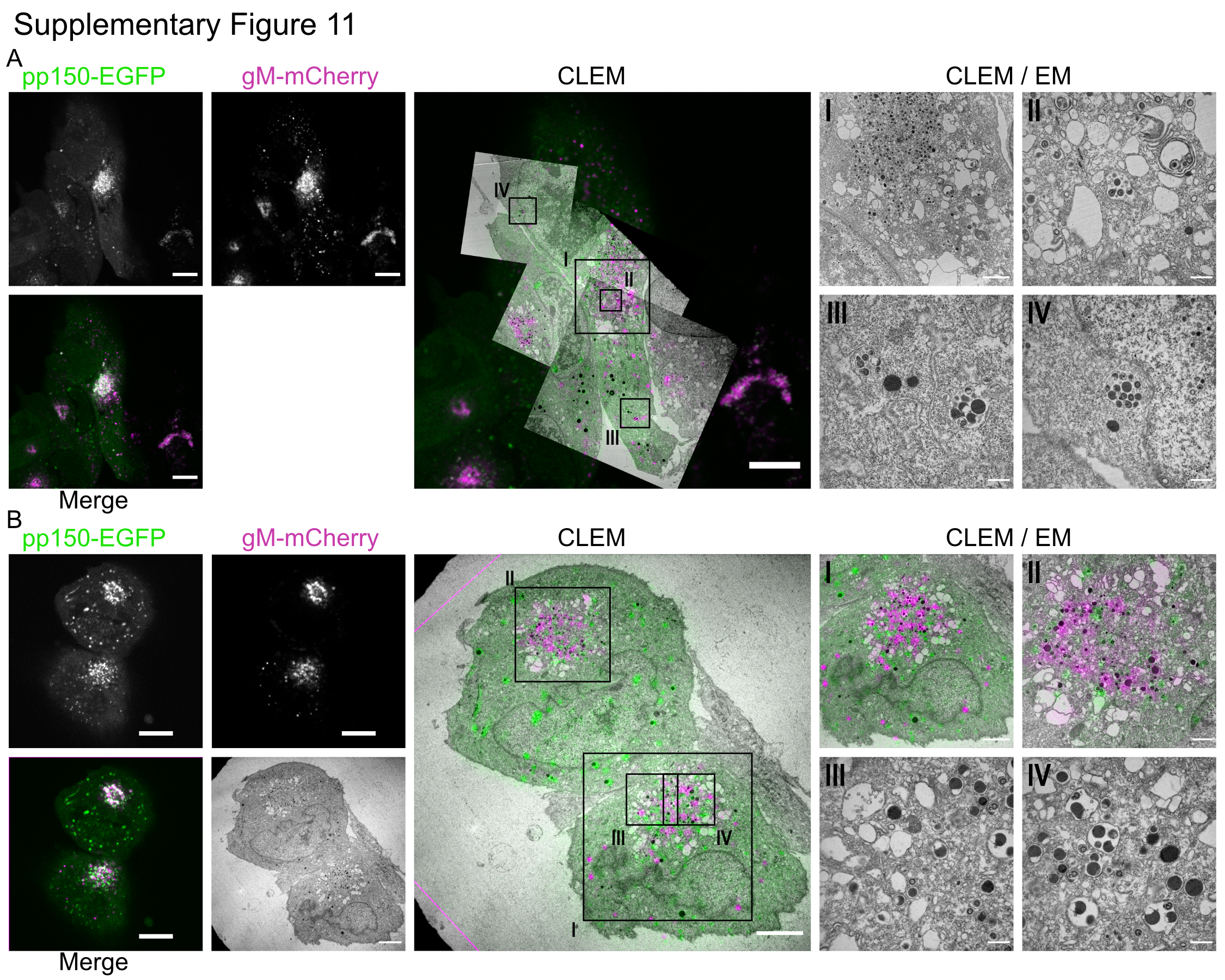

Supplement: S11 Fig — HFF-cells were infected as described in Fig 6D and 6E. CLEM reveals large cytoplasmic vesicles positive for viral proteins to be MViBs. (TIF) [file ppat.1010575.s011.tif]
