# Supplementary material for: Intermittent bulk release of human cytomegalovirus
Source: PLoS Pathog. 2022 Aug 4;18(8):e1010575. doi: 10.1371/journal.ppat.1010575 (PMC9352052; doi:10.1371/journal.ppat.1010575)
Supplement: S1 Text — (DOCX) [file ppat.1010575.s013.docx]

**Supplementary Text 1 (S1 Text)**

The video files can be downloaded from Dryad/Zenodo [1]:

https://datadryad.org/stash/dataset/doi:10.5061/dryad.gtht76hpt

https://zenodo.org/record/6611135#.YrVm4ufgphE

**Supplementary Video 1. SBF-SEM of an area between the cell surface and the growth substrate.** The video shows a subset of planes from the dataset described in Fig 1 rendered as a video. Infection conditions are as described before. The signal was inverted to resemble TEM contrast. Shown is a large invagination below the cell at the growth substrate.

**Supplementary Video 2. 3D-rendering of an MViB from SBF-SEM data.** In this video an MViB from the SBF-SEM dataset described in Fig 2 is rendered in 3D. The yellow surface marks the limiting membrane of the multivesicular structures. The contents are rendered as surfaces in different colors to show the heterogeneity of the MViB cargo. Virions are rendered in dark green, dense bodies in cyan, and other vesicular material in magenta. Scale bar indicates 600 nm.

**Supplementary Videos 3A-B. SBF-SEM rendering of infected HFF cells.** The video shows an excerpt from the dataset described in Fig 1. HFF cells were infected with an MOI of 3 and fixed 4dpi. **3A** Overview rendering of the whole SBF-SEM dataset of the cells shown in Fig 1 and 2. **3B** A group of prominent virus-filled MVBs is highlighted by a surface rendering. Several more MVBs are present in the cell.

**Supplementary Video 4. Stack of an MViB and associated immature particles.** This video shows an MViB from the SBF-SEM dataset shown in Fig 1. Cells were infected and treated as described before. White triangles indicate immature, non-enveloped particles in close proximity or directly associated with the multivesicular structure next to the nucleus. Scale bar indicates 0.2 µm.

**Supplementary Video 5. Multi-perspective 3D rendering of volumetric time-lapse microscopy data of HCMV release.** HFF cell, infected with HCMV-pp150-EGFP-gM-mCherry as described in Fig 3A. The video shows several perspectives on how a large MViB positive for pp150-EGFP (green) and gM-mCherry (red) traverses the cytoplasm and fuses with the plasma membrane. The first seconds show the 3D video, followed by a split-screen part of three different perspectives. A spotlight effect (circle) highlights the same body in all three parts. In the left third, the MViB is followed by a moving section parallel to the growth substrate, through the volume on its way downwards to the lower cell surface. In the middle part, the body is followed as a 3D rendering through the cell. The camera angle moves to keep the body visible as well as possible. The last third shows how the MViB fuses with the plasma membrane in a static cross-section. Due to the optical setup of the lattice-light-sheet microscope (See Fig 3A), the grid added by Arivis 4D (Arivis AG, Rostock, Germany) is tilted 30° respective to the real physical orientation of the cell in the microscope.

**Supplementary Videos 6-7. Live-cell long time-lapse spinning-disk microscopy videos.** HFF cells were infected with HCMV-pp150-SNAP-gM-mScarlet-I at an MOI of 1. At 72 hpi, cells were stained for pp150-SNAP and imaged live by spinning-disk microscopy. 8-micrometer stacks in 1-micrometer increments were acquired every 40 minutes. The plane shown is the section of the cell closest to the coverglass. Cells can be seen to release virus particles in short intermittent bursts over several hours, indicated by the white arrowheads. pp150-SNAP labeling is shown in green and gM-mScarlet-I label in magenta. The time format is hh:mm.

**Supplementary Video 8. 3D rendering of immunofluorescence data.** In this video, the IF dataset from S6 Fig is rendered in 3D. The coloring scheme is the same as in S6 Fig. The 3D rendering shows the 3-dimensional correlation between the molecules.

**Supplementary Video 9. Lattice light-sheet microscopy of HCMV bulk release.** HFF-CD63-pHluorin were infected with HCMV-TB40-pp150-SNAP-gM-mScarlet-I at an MOI of 1. At 4 dpi the cells were live-stained for pp150 with SNAP-Cell-SiR and imaged by lattice light-sheet microscopy. The two rows of panels show sections of the same cell in different heights. The AC, EVAs and EVA-formation events are marked in the video.

**References**

[1] Flomm FJ, Soh TK, Schneider C, Wedemann L, Britt HM, Thalassinos K, Pfitzner S, Reimer R, Grünewald K, Bosse JB. "Intermittent Bulk Release of Human Cytomegalovirus" Associated Data; 2022 [cited 2022 May 30]. Database: Dryad [Internet]. Available from: <https://doi.org/10.5061/dryad.gtht76hpt>
