# Supplementary material for: Intermittent bulk release of human cytomegalovirus
Source: PLoS Pathog. 2022 Aug 4;18(8):e1010575. doi: 10.1371/journal.ppat.1010575 (PMC9352052; doi:10.1371/journal.ppat.1010575)
Supplement: Attachment — Submitted filename: PbP_response_Flomm_et_al_2022.pdf [file ppat.1010575.s014.pdf]

Point-by-Point Response to the Reviewer's Comments

We thank the reviewers for their comments and suggestions. Here, we provide a substantial revision, addressing all reviewer comments. Importantly, we performed additional experiments to confirm and strengthen the results and to address the major concerns regarding the identity of the virus-containing multivesicular structures that mediate bulk virus exocytosis. To this end, we have also rewritten the manuscript to clearly discriminate these structures, which we now term multiviral bodies (MViBs) from cellular multivesicular bodies (MVBs in *sensu stricto*). Finally, we changed the title to avoid any misconceptions regarding the use of the term MVB. The proteomics data deposited are available via ProteomeXchange with identifier PDX023444 and with the following credentials:
Username: reviewer_pxd023444@ebi.ac.uk
Password: Bzndr0fO

Editor comment:

Dear Prof. Dr. Bosse,
Thank you very much for submitting your manuscript "Egress of human cytomegalovirus through multivesicular bodies" (PPATHOGENS-D-21-01612) for consideration at PLOS Pathogens. As with all papers peer reviewed by the journal, your manuscript was reviewed by members of the editorial board and by three independent peer reviewers. Based on the reports, we regret to inform you that we will not be pursuing this manuscript for publication at PLOS Pathogens.
The reviewers believe the work to be of importance and the topic to be of interest. However, Reviewers 1 and 3 have raised a number of significant concerns that will require substantial experimentation. These include providing evidence that the structures called MVBs are indeed those compartments, improved identification that viral particles purported to be undergoing secondary envelopment are actually such particles, evidence that blocking MVB formation impacts detection of EVAs, and comparing the released viruses to purified virus-containing MVBs from infected cells using mass spectrometry. In addition, there were several other concerns that are described in the reviews below.
Taking into consideration the number of concerns raised, we regret that we will not be able to accept this manuscript for publication. However, if you decide to address the reviewers' concerns and resubmit, please make sure to carefully address each concern. When resubmitting the manuscript as a new manuscript, please state in your cover letter that the manuscript was evaluated before and provide a point-by-point response to the reviewers' comments.
The reviews are attached below this email, and we hope you will find them helpful. We are sorry that we cannot be more positive on this occasion. We very much appreciate your wish to present your work in one of PLOS's Open Access publications.
Thank you for your support, and we hope that you will consider PLOS Pathogens for other submissions in the future.

Part I - Summary
Please use this section to discuss strengths/weaknesses of study, novelty/significance, general execution and scholarship.

Reviewer #1:
This manuscript by Flomm et al entitled, "Egress of Human Cytomegalovirus though multivesicular bodies" follows up recent reports that found enveloped virus particles inside multivesicular bodies (MVBs) but were unable to ascertain whether the detected particles reflect a productive or a degradative pathway. Using 3D correlative fluorescence and EM (CLEM), the authors contend that HCMV secondary envelopment occurs at MVBs and that virus-filled MVBs traverse the cytoplasm and release virions in bulk at the plasma membrane, resulting in extracellular virus accumulations. They conclude that MVBs represent a novel bona fide HCMV egress pathway and suggest that the use of divergent egress pathways contributes to the observed broad host range exhibited by these viruses. Three aspects to which little attention is given are (1) validation that the structures called MVBs are actually those

compartments; (2) clear identification that particles purported to be undergoing secondary envelopment are actually such particles; and (3) evidence that blocking MVB formation impacts detection of EVAs.

Note: For clarity, we moved the reply to comments of Reviewer #1 from the Summary section into Part II - Major comments and cover there all three raised aspects.

Reviewer #2:
The manuscript submitted by Flomm et al., described the accumulation of CMV in multivesicular bodies and the subsequently release into extracellular EVA (extracellular accumulation). The author group used a 3D-CLEM to observe the fluorescence-labeled virus to trace the intracellular morphogenesis process and the viral egress pathway. It was reported in the literature that is involved CD63 associated MVB is involved in CMV cytoplasmic morphogenesis. This study then focused on the localization of CD63 close to cell membrane. The technique used in this study is interesting. Whether this finding can explain the virion diversity remained to be explored.

Reviewer #3:
The manuscript “Egress of human cytomegalovirus through multivesicular bodies” by Flomm et al. utilize state of the art electron and fluorescence microscopy techniques to assess the role of virus-containing multivesicular bodies in the egress of hCMV. The authors detected extracellular viral accumulations and suggest that these are likely to be the result of viral egress through the virus-containing multivesicular bodies. The work present in this manuscript suggests that hCMV uses the virus-containing multivesicular bodies as a pathway for viral envelopment and egress. This is novel egress mechanism that have not been seen in the well-studied egress processes of alphaherpesviruses and thus of interest to many virologists. The authors provide strong evidence to support their model however several changes can improve the paper

We thank Reviewers #2 and #3 for appreciating the technical aspects of our approach and clear novelty of the described and strongly supported egress mechanism.

Part II – Major Issues:
Key Experiments Required for Acceptance. Please use this section to detail the key new experiments or modifications of existing experiments that should be absolutely required to validate study conclusions. Generally, there should be no more than 3 such required experiments or major modifications for a "Major Revision" recommendation. If more than 3 experiments are necessary to validate the study conclusions, then you are encouraged to recommend "Reject".

Reviewer #1:
Three aspects to which little attention is given are (1) validation that the structures called MVBs are actually those compartments; (2) clear identification that particles purported to be undergoing secondary envelopment are actually such particles; and (3) evidence that blocking MVB formation impacts detection of EVAs.

Reg. 1) We needlessly caused ambiguity by using "MVBs" in the title and abstract instead of a distinctive term. In the revised manuscript we now use the term "multiviral bodies (MViBs)" to describe the phenotype of a large vesicle filled with a large number of virus particles. We do not want to claim that MViBs are identical to the MVBs found in uninfected cells.
In the manuscript, we show that human cytomegalovirus (HCMV) capsids can envelop in large numbers at large vesicles resulting in exocytic compartments containing tens to hundreds of virions that are subsequently released intermittently into extracellular viral accumulations (EVAs). As we find numerous EVAs on more than 85% of all infected cells comprising thousands of virus particles per cell, bulk release seems to be relevant and a so far overlooked HCMV egress pathway. While the initial biogenesis of these virus-filled compartments is important, it is largely out of the scope of this work.

We added several statements that we do not wish to claim any parallels between cellular MVBs and MViBs (Lines 113-114, 303-305, 333-334, 403-404). Finally, we changed the title to better state the key findings of this manuscript to “Intermittent Bulk Release of Human Cytomegalovirus”.
Still, we followed the reviewers’s guidance and used immuno-EM as well as a panel of MVB/exosome inhibitors in an attempt to delineate the biogenesis of MViBs (see below). However, as known, HCMV extensively remodels the cellular secretory system over days of infection which makes it extremely challenging to use classical cellular markers to identify cellular organelles which have been co-opted by the HCMV proteome.[1,2,3]

Reg. 2) Block face scanning EM results in almost classic thin section EM representations of herpesvirus capsids as round to hexagonal shells, filled with a bar-shaped or dot-like density (depending on their orientation in the imaging plane,) which stems from the condensed viral DNA after aldehyde fixation. In contrast, high-pressure freezing and freeze-substitution in the presence of uranyl acetate leads to almost black particles in EM[4]. Some examples for aldehyde-fixed herpesvirus particles can be found in [5] and [6], and there are numerous more since this preparation technique has been used since the advent of electron microscopy. To illustrate that virus particles can be readily identified in 3D-EM stacks of infected cells, we have added sup. Fig. 1 depicting virus particle morphogenesis throughout various stages of the viral life cycle.

Reg. 3) As mentioned above, we do not wish to claim that MViBs and cellular MVBs have the same identity, nor that they are of the same descent. Still, we have investigated the susceptibility of MViBs and their resulting EVAs to a panel of three known inhibitors of MVB/Exosome formation (Sup. Fig. 9). We found that only Tipifarnib, a farnesyltransferase-inhibitor, reduces EVA numbers and accordingly viral titers up to one hundred-fold. However, Tipifarnib also affected the late viral gene pp150, which is part of the inner tegument. Further studies are, therefore, needed to study the biological identity of MViBs.

Note: For clarity, we moved the following comments of Reviewer #1 from the Summary section:

1. Figure 2: The results are accurately described in the text but the conclusion is not compelling: A limiting factor in previous studies was the ability to demonstrate secondary envelopment events in the vesicles identified as MVBs. Here, the authors correlate specific labels for capsids (pp150) and viral membranes studded with a transmembrane viral protein (gM) with volumetric EM data of whole infected cells to detect EVAs (extracellular virus accumulations) at late times below the cell, suggesting the existence of exocytosis hotspots. As they indicate, they found that infected cells accumulated a plethora of materials of different sizes and plasma membrane invaginations that might reflect endo- or

---

[1] Close, William L., et al. "Infection-induced changes within the endocytic recycling compartment suggest a roadmap of human cytomegalovirus egress." *Frontiers in microbiology* 9 (2018): 1888.

[2] Moorman, Nathaniel J., et al. "A targeted spatial-temporal proteomics approach implicates multiple cellular trafficking pathways in human cytomegalovirus virion maturation." *Molecular & Cellular Proteomics* 9.5 (2010): 851-860.

[3] Das, Subhendu, and Philip E. Pellett. "Spatial relationships between markers for secretory and endosomal machinery in human cytomegalovirus-infected cells versus those in uninfected cells." *Journal of virology* 85.12 (2011): 5864-5879.

[4] Buser, Christopher, et al. "Cytomegalovirus primary envelopment occurs at large infoldings of the inner nuclear membrane." *Journal of virology* 81.6 (2007): 3042-3048.

[5] Bodaghi, Bahram, et al. "Entry of human cytomegalovirus into retinal pigment epithelial and endothelial cells by endocytosis." *Investigative ophthalmology & visual science* 40.11 (1999): 2598-2607.

[6] Chaumorcel, Magali, et al. "Human cytomegalovirus controls a new autophagy-dependent cellular antiviral defense mechanism." *Autophagy* 4.1 (2008): 46-53.

exocytosis of EVAs. The authors' effort to provide images of vesicles dually labeled with viral proteins in the context of the cell volume is convincing (panels C-F).

However, (a) there is no evidence provided in the figure that the structures associated with virus-like particles (VLPs) are MVBs. Dual labeling with a MVB marker is needed here.

As discussed above, our original intent was not to claim that the provenance of MViBs and MVBs is the same. However, to support our conclusion that MViBs identified in EM are the CD63 positive structures that lead to EVA formation in our TIRF-assay, we performed anti-CD63 immuno-EM (Sup. Fig. 8). While structural conservation in Tokuyasu cryo-sections for immune-EM is partially compromised for preserving antigenicity, we were still clearly able to confirm that MViBs were positive for CD63.

(b) Also, panels G and H do not provide convincing images of unenveloped immature VLPs expected to be associated with MVB compartments near the nucleus (i.e., point of origin). Again, dual immunogold labeling would be helpful here.

As mentioned in the general section above, DNA-containing C-capsids are readily identifiable due to their conserved phenotype in aldehyde-fixed EM samples. We added sup. Fig. 1 to illustrate our point.

(c) Although panel H enlarges a region with smaller VLPs, the image in panel G from which it is derived contains (as the authors admit) a heterogeneous collection of particles with diameters significantly larger than that of virus particles. As VLPs appear to be a minor component, it is difficult to assess confidently that the compartment comprises a productive element of replication fated to be delivered to the cell surface.

HCMV has been shown to produce large numbers of exosomes and other non-capsid-containing vesicles that were reported to play a role in host modulation[7,8].These components can also be found in virus stocks[9]. The MViBs described in our manuscript contain a large number of such particles, including dense bodies (enveloped tegument-only particles) and virus particles. Our data shows that very similar particle compositions can be found in extracellular EVAs, and we added a quantitative comparison of MViB to EVA contents to illustrate this point (Fig. 2E). We also added Sup. Vid. 2, which shows a 3D rendering of an MViB with its content color-coded as well as Sup. Vid. 4 that illustrates viral particles budding into an MViB filled with mixed contents.
Moreover, we present live-cell microscopy data indicating that large bodies positive for pp150 and gM and shown in 3D-CLEM to be MViBs are transported to the plasma membrane. TIRF microscopy indicates that these MViBs fuse with the plasma membrane and result in EVAs (Fig. 4). Therefore, our data demonstrate that MViBs with all their mixed cargo are indeed undergoing exocytosis.

2. Lines 166 to 170: Several aspect of the data placed in Supplementary material should be presented in the main text instead. Examples are (a) "vMVBs" containing several hundred VLPs, especially if the VLPs exhibit the expected range of size, morphology and state of maturity and (b) vMVBs in cells infected with wild-type HCMV. This should be accompanied by a quantitative assessment of frequency of detection in the cell population.

---

[7] Pepperl, Sandra, et al. "Dense bodies of human cytomegalovirus induce both humoral and cellular immune responses in the absence of viral gene expression." *Journal of virology* 74.13 (2000): 6132-6146.

[8] Mohammad, Abdul-Aleem, et al. "Human cytomegalovirus microRNAs are carried by virions and dense bodies and are delivered to target cells." *The Journal of general virology* 98.5 (2017): 1058.

[9] Talbot, P., and June D. Almeida. "Human cytomegalovirus: purification of enveloped virions and dense bodies." *Journal of General Virology* 36.2 (1977): 345-349.

We added more examples of MViB imaging stacks as supplementary videos (Sup. Vid. 2-4). Unfortunately, it is very challenging to quantify MViB frequencies in infected cells as they are highly transient. However, EVAs can be readily quantified as they are relatively stable and represent the endpoint of this process. As shown in Figure 1B, we now quantified EVAs occurrence with labeled and WT virus and got very similar results, arguing that EVA formation is not an artifact of fluorescent protein tagging.

3. Figure 3B: It is difficult to assess how much of the correlation is coincidental and how much is actual capid-envelope co-localization. It is even more difficult to be confident that viral protein-MVB association reflects “vMVB” movement. Also, the authors do not address the apparent dissolution of the structure indicated by the arrowhead between minutes 5:29-6:51, followed by the reappearance of a structure where the fluorescence overlaps but is not merged from minutes.

The reviewer makes a good point that it is very challenging to depict complex 4D imaging data in 2D figures. For this reason, we added the Sup. Vid. 5 that shows a 3D rendering from three different perspectives to make it clearer that capsid-envelope signals are closely associated over time such that it is very likely that signals indeed are co-transported and do not overlap stochastically. Of note, we would like to add that live-cell data as presented here has the advantage that it can provide temporal correlation of signals, thereby providing a much higher fidelity compared to fixed and static immunofluorescence (classic colocalization). In reference to the specific event referred to by the reviewer, what seemed as the dissolution of the vesicle was actually due to it moving in 3D. We modified Fig. 3B to now depict maximum intensity projections of a 3D stack to account for the movement of the objects in 3 dimensions.

The authors state in the text (line 190) that “release events varied in fluorescence intensity, which is consistent with our observation that vMVBs were very heterogeneous in size and content.” However, that does not apply to the arrowhead in the figure, which follows a single “vMVB” cluster.

This note is in regard to the Fig. 3C as well as Sup. Vid. 6 and 7 which show numerous EVA formation events which vary largely in their size. The respective figure references are now given in the text.

4. Lines 196-198: To test their assumption that confirm that the observed bulk release events are induced by fusion with the plasma membrane, the authors created a cell line stably expressing a CD63-pHluorin fusion construct as they have data indicating that CD63 is enriched on vMVBs membranes but not on the virions themselves. This data should be provided in the supplementary material.

We apologize for this confusion. The data referred to in this sentence is presented in the next paragraph. We clarified this in the text. Fig. 5 shows CD63 enrichment on the enwrapping MViB’s membranes, while our MS data for purified virions does not indicate the presence of CD63 on enveloped virions. In addition, we provide CD63 immune-EM data in in sup. Fig. 8 to support the respective statement in the text.

5. Line 911-912, Figure S5B: The authors conclude that the CD63 signal correlates with gM and gB signals. While it clear correlates with the gB signal, it is less clear that the gM signal is specifically (rather than coincidentally) present.

We added Sup. Vid. 8 to illustrate that gB and gM both colocalize in large vesicular structures positive for CD63.

6. Figure 4, lines 215 to 220: A key conclusion in the paper, i.e., that EVAs are formed following fusion of vMVBs and the plasma membrane, relies on what is shown in this figure. While a case could be made that some of the arrowheads in the figure show post-fusion EVAs, others are less evident. Also, it is not clear that the gM-mScarlet-I and the pp150-SNAP are denoting the same particles.
Another problem is that the text states several conclusions that are not readily evident from the figure: e.g., (1), “The gM signal increased directly before the fusion event” (signaled by CD63 flash) --- this is

evident in panel C. However, (2), “and decreased as vMVBs relaxed into a flattened patch.”: The conclusion that the vMVBs relaxed is an assumption based on the premise that the gM signal represents a virion. As indicated above, this is not convincing.

We revised Figure 4 and added a panel illustrating the pp150 signal traces through EVA formation. We modified the text accordingly to make our point clearer (Lines 234-236).

(3), “The exocytosed material emitted a continuously elevated signal. These results are consistent with CD63 not beingincorporated into virions (Fig. 5).” Here, a conclusion is drawn from the absence of a signal. Although the authors contend that their conditions preserve signals over long periods, this support for their conclusion is not compelling.

We wanted to make the point that the CD63 signal diminishes more strongly after plasma membrane fusion before reaching a plateau, while pp150 and gM signals remain constant at higher levels. Since all three markers plateau well above background levels, as depicted in Fig. 4C, and stay constant, signals are indeed preserved and not bleached.

(4), “The transition from vMVBs to EVAs took much longer than the actual fusion event, suggesting that fusion pore expansion and cargo expulsion were slow.” It seems equally plausible that the transition took longer than the authors anticipated because other explanations exist.

We agree that other reasons might exist and removed the statement as it was merely an observational side note and not essential to the results of our work (Line 241-242).

7. The authors performed mass spec of gradient-purified extracellular virions to assess virus versus exosome formation. They found markers of Golgi-to-endosome trafficking, early endosomes and exosomes and concluded that HCMV might use a mix of membranes originally originating from Golgi- and endosomal membranes for secondary envelopment to generate vMVBs. Isn’t a more straightforward conclusion simply that HCMV might use a mix of membranes originally originating from Golgi- and endosomal membranes for secondary envelopment of the particles that become virions?

We apologize for the confusion. This is precisely what we meant. We modified the text accordingly (Lines 253).

8. Lines 233-234; lines 310-313; 327-334: The Turner et al paper referred to by the authors concluded that CMV uses the existing host exosome machinery for maturation and egress based on their finding that knock down of several proteins identified in a proteomic analysis of exosomes reduced HCMV production significantly. However, in contrast to the authors finding, the Turner report concluded that the classical exosome markers, such as CD63 used here as a marker, were not associated with the viral structures and, instead, indicated that HCMV co-opted a novel nexus of exosome components.

We state exactly this point. Our data indicates that CD63 is on the MViB limiting membrane but not on the virion (Lines 267-270).

Moreover, the Turner group found evidence for the novel participants in gradient fractions enriched for HCMV virions examined as early as 24 hr post-infection, which is significantly sooner that the authors’ finding of small numbers of EVA “hot spots” 72 to 94 hr following viral protein expression.

HCMV does not produce virions after 24 hpi, and we could not find the referred part in Turner et al. 2020. Instead, most of the proteomic data from the said publication were generated at 5 days post-

infection, which is significantly later than the time range in which we investigated EVA formation.[10] Does the reviewer potentially refer to the IE1 IFs that were done in Turner et al. at 24 hpi?

The authors mainly focus on the supporting aspects of the Turner report; they should in addition include some discussion of the important differences especially since they mention the existence of contradictory evidence in the field regarding CD63 in HCMV virus production (lines 310-313).

As mentioned above, we do not see a discrepancy in our findings in relation to the work of Turner et al. Our data on the dynamics of virion release also goes significantly beyond the Turner report. We expanded in the discussion on the role of exosome pathway factors, such as CD63, and their role in HCMV assembly and egress (Lines 359-367, 371-374).

We would like to thank reviewer #1 for carefully and critically reading our manuscript. Her/his questions clearly helped in improving the manuscript by adding previously missing data to support the conclusions and in presenting our data and conclusions in a better way.

Reviewer #2:
(No Response)

Reviewer #3:
I am not sure how the Mass spec. experiment had contributed to this study and to previously published Mass spec. experiments. I suggest either removing the experiment or further investigation into the Mass Spec results. It will be nice to compare the results of the released viruses to purified virus-containing multivesicular bodies that can be purified from infected cells. This is a challenging experiment but will contribute to the strength of the suggested hypothesis.

We agree with Reviewer #3 that the included mass spectrometry data is largely confirmatory since Turner et al. 2020 report similar findings, although our data was generated using strain TB40 while Turner et al. used AD169.[11] However, the data also serves as a control to confirm that our experimental system is comparable to other reports. We then build on it and show that MViBs mediate the bulk-release of virus particles leading to the formation of EVAs. For this reason, we would like to keep the data in the manuscript but can, of course, remove it if the reviewer insists. We added Sup. Fig. 6 to illustrate our MS results better.
We also agree that comparing the proteome of MViBs to the released virus would be a nice optional addition to the manuscript. However, as also stated by this reviewer, own preliminary results point in the direction that it is extremely challenging to make sure that the resulting "vMViB-preparations" are pure and constitute the organelles that we described *in situ*. We, therefore, would like to ask not to include this analysis as it would be out of the scope of this study.

Part III – Minor Issues: Editorial and Data Presentation Modifications.
Please use this section for editorial suggestions as well as relatively minor modifications of existing data that would enhance clarity.

Reviewer #1:

Note: For clarity, we moved following comment of Reviewer #1 from the Summary section:

1. Figure 3 legend: typo: HCMV-pp150-SNAP-gM-mScarlet-I

---

[10] Turner, Declan L., et al. "The host exosome pathway underpins biogenesis of the human cytomegalovirus virion." *Elife* 9 (2020): e58288.

[11] Turner, Declan L., et al. "The host exosome pathway underpins biogenesis of the human cytomegalovirus virion." *Elife* 9 (2020): e58288.

We corrected this error.

Reviewer #2:

1. Because the bulk release of CMV at the cell periphery is a major finding of this manuscript, it is suggested to draw a line to indicate cell margins in the data, eg. Fig 1C and Fig. 3C.

We thank the reviewer for the suggestion. However, the cell boundary at which viral release happened is in these images the lower plasma-membrane, which is from the viewing angle of the inverted microscope a plane and therefore cannot be marked by a line.

2. Fig. 4B is difficult to interpret the fluorescent signals. Fig. 4C, the description about the fusion event is hard to follow.

We have modified the figure and text to make this clearer (Lines 234-236).

3. Sup Table 1 is difficult to read, a pathway analysis or grouping of the proteins according to their function may help the readers to have an overview.

We have added a pathway analysis (Sup. Fig. 6).

4. In the discussion section, Line 308, the controversy about CD63 function in CMV replication in previous studies is mentioned. It would be better to discuss a little bit about the possibility that causes the differences.

We have extended the discussion regarding this point (Lines 359-367, 371-374).

5. Line 133, The description in the text, Fig. 1C, 1B showed up before 1A

We have corrected this error. However, the subfigure A is in most of the main figures a schematic, which guides the reader through the images and is therefore not referenced in the main text.

6. Line 186, needs more explanation for “EVAs seemed to also attach to the growth support”.

We modified the manuscript accordingly (Lines 206-207)

Reviewer #3:

1. The resolution of the images (at least in the PDF format) are not sufficient to clearly decide many of the suggested observations, however the supporting movies are much better. At least in figure 3 I suggest decreasing the number of time points and increasing the size of the representative images

The compression used in the PLOS Submission system to generate .pdfs, unfortunately, reduces image quality significantly. The high-resolution figures are also available in our preprint on BioRxiv (https://www.biorxiv.org/content/10.1101/2020.12.31.424954v5.abstract). Final figure quality will allow zooming in without restricting the number of time points.

2. The authors change the MOI among the different experiments with no explanation, while I assume only minor differences among MOI 1 to 5, it would be nice if the authors will explain these differences and for the experiment in figure 4 the MOI is not included.

We have added a discussion about MOIs used to the manuscript in the Materials and Methods section under the subheading Cells and Viruses (Lines 447-451)

3. The order of figure 5B panels is confusing and should be as usually presented as one line for each image.

We have modified the Figure accordingly.

4. Lines 515-517 there are two 7dpi in the two sentences, please rewrite for clarity.

We corrected this error.
