# Supplementary material for: Intermittent bulk release of human cytomegalovirus
Source: PLoS Pathog. 2022 Aug 4;18(8):e1010575. doi: 10.1371/journal.ppat.1010575 (PMC9352052; doi:10.1371/journal.ppat.1010575)
Supplement: Attachment — Submitted filename: Authors Response.docx [file ppat.1010575.s015.docx]

**Point by point response:**

We sincerely thank all reviewers for their critical reading of our manuscript and their insightful and fair suggestions. Below is our point by point response to the reviewers’ comments (highlighted in light blue).

Please note that the resolution and quality of the figures had to be reduced due to file size limits in the editorial management system. You can find the all figures, supplementary figures and videos via these links:

All Figures and Videos:

<https://syncandshare.desy.de/index.php/s/6x4ciHeD982Yk9Q>

Main Figures Full Size:

<https://syncandshare.desy.de/index.php/s/SB8rymWMkEmLqYW>

Supplementary Figures Full Size:

<https://syncandshare.desy.de/index.php/s/246YDFgNePHnPk7>

Supplementary Videos:

<https://syncandshare.desy.de/index.php/s/PPGtqfnXSqTSL4F>

The proteomics data deposited are still available via ProteomeXchange with identifier PDX023444 and with the following credentials:

Username: reviewer_pxd023444@ebi.ac.uk

Password: Bzndr0fO

**Part I - Summary**

**Reviewer #1:**

The manuscript by Flomm et al describes studies using sophisticated fluorescence and electron microscopy imaging techniques to characterize structures within HCMV-infected cells associated with virion morphogenesis and egress. The authors describe large collections of virus particles at the exterior periphery of late-state infected cells, which they term extracellular viral accumulations (EVAs). They also describe vesicles within the cells containing wildly varying numbers of enveloped viral particles (virions and dense bodies), which they term multiviral bodies (MViB). Imaging data appear to show that MViB are a site of secondary envelopment and that fusion between the limiting membrane of the MViB and the cellular plasma membrane releases enveloped viral particles in bunches (i.e. EVAs). The identification of host cell secretory proteins associated with MViBs is consistent with the general herpesvirus model of secondary envelopment/morphogenesis at compartments derived from the cell secretory pathway.

Overall, the data are of high quality and the interpretations seem reasonable. However, I come away with the sense of not having learned much about HCMV egress beyond; 1) seeing a more detailed picture of the morphologic range of secondary envelopment compartments, and 2) seeing that these compartments can be larger and contain more virions than is commonly depicted in schematic diagrams of the “assembly compartment” (AC). The authors seem to draw a distinction between the MViB and the traditional description of the AC. But, given the drastic remodeling of the secretory/endosomal compartments in HCMV-infected cells, and the apparently wide variation in the size and viral contents of the so-called MViBs, it could be that the previously named ACs might be a type of MViB. Are the endosomal/exosomal pathway markers associated with MViBs specifically not associated with the traditional “AC”? That said, these studies lay an excellent technical framework for the study the specific mechanism of viral and host cell factors in virion morphogenesis and egress. – Brent Ryckman, University of Montana.

We thank reviewer #1 for his supportive evaluation of our work. Concerning the nature of the assembly complex (AC), there might be some misunderstanding: The HCMV AC is often described as a large donut-shaped organelle based on immunofluorescence data. However, transmission electron microscopy data (TEM) shows that the AC consists of a complex juxtanuclear accumulation of vesicles of varying size and identity, which are centered around a Golgi-derived MTOC [1–4]. Viral secondary envelopment is mostly described as capsids budding into individual small vesicles in the ACs center, which presumably then egress individually and continually. However, actual live-cell data for this model is missing for HCMV [3]. We now describe “grouped” envelopment resulting in multivesicular structures and show their release in bulk from living cells. We, thereby, define a functional role to a novel type of vesicle that is part of the AC.

**Reviewer #2:**

Manuscript: Intermittent Bulk Release of Human Cytomegalovirus (PPathogens –D-22-00174)

Authors: Flomm, F., Soh, TK, Schneider, C., et.al.

This revised manuscript provides evidence from several sophisticated imaging technologies that human cytomegalovirus (HCMV) assembles in large cytoplasmic structures (MViBs) and infectious virus in these structures is release following an exocytosis and fusion with the PM. These authors argue this route of virus release opens an area of research to further define mechanisms of virus diversity. The version I received had been previously reviewed and had extensive changes in the text in response to the previous review.

The data provided in this paper clearly supports the hypothesis on line 180 but does not provide definitive data for this mechanism of egress. The lack of either loss or gain of function studies to test this hypothesis are a major limitation of this study because it leaves the reader with a novel mechanism for HCMV egress but no definitive conclusion. This is probably best summarized by the honest assessment by the authors of their data (lines 288-292). Finally, this study has no data that in any way would connect the interpretations of the imaging data with the release of virions with specific cell tropism. This may be a part of their hypothesis but it is not clear how this can be integrated into the data or discussion of the data that was presented in this manuscript.

We thank reviewer #2 for his critical comments. These are valid points: 1) We share this reviewer’s frustration that we were not able to present data from loss/gain of function experiments to further support our conclusions. After the first round of revisions, we tested a wide range of reported MVB/exosome inhibiting substances in an effort to pinpoint the pathway and potential molecular factors involved. The idea was that we could use loss/gain of function assays in the next step to verify their involvement. Unfortunately, we were not able to clearly dissect the involved mechanism by this approach, and future studies are needed. However, we now also include data using BAC-derived Merlin with restored UL128 and RL13 expression that produces MViBs while EVA production is diminished. These data suggest that MViB production is a general phenomenon of HCMV morphogenesis but that strain-dependent differences exist in their release (see new Fig. 6, Sup. Fig. 10-11, lines 336-345, as well as a discussion of the data in lines 383-390, 403-411). Therefore, we believe that this manuscript is of interest to a wider audience as it describes significant data suggesting an important role for a so-far neglected egress mechanism of HCMV. 2) We also agree that we do not present data that directly connects MViB-mediated egress with tropism. We merely suggest that differing egress pathways might enable the production of virions of divergent composition and that this possibility should be investigated in future work.

Specific queries:

1) pp150 is associated with the capsid, but it is not a capsid protein and can be found in infected cells without any spatial associations with capsids or capsid proteins (line 149).

We apologize for the misleading wording and corrected the sentence (lines 187-188).

2) Line 204. It is unclear what “relaxed into patches” means to this reviewer. I can understand a spatial accumulation but “relaxing” is not a very clear descriptor.

We revised the wording for clarity (lines 156, 243, 248, 282).

3) The resolution of some of the images that rely on the fluorescent protein tagged virus was such that the reader is really dependent on where the authors put their arrow in the figures. This could be improved so that the reader can evaluate the data without total reliance on the placement of an arrow.

The lattice light-sheet microscope used for volumetric live-cell imaging trades minimal cytotoxicity, extremely fast volumetric imaging, and isotropic resolution with a reduced x-y resolution of about 300 nm. We can, therefore, not improve the resolution of the fluorescence images. However, we believe the main issue is the apparent image compression of the BioRxiv and PLoS Pathogens submission system. We tried to optimize the submitted pdf in this resubmission such that fewer artefacts are induced. For this resubmission, we included links where the full size figures and videos can be downloaded from servers of the german electron synchroton DESY at the top of this author’s response and in the editorial management system.

4) The term of MViB is an obvious compromise. However, what endomembranes contribute to these structures? Can they be isolated and analyzed? This question is central to their hypothesis, and as a result, this manuscript could be viewed as a preliminary report.

This question has also come up in the previous round of review as a suggestion for a potential experiment, and we agree that this data would be of great interest. However, preparing MViBs and ensuring identity, quality, and purity in quantities sufficient for proteomics is a highly challenging task with no established protocols that could be applied in the given timeframe of this revision.

**Reviewer #3:**

This study uses advanced microscopy and biophysics based approaches, e.g., live-cell lattice light-sheet microscopy (LLSM) and three-dimensional correlative light and electron microscopy (3D-CLEM), to study how HCMV virions bud from infected cells during late morphogenesis and exit. In response to previous reviewers, the authors stress that they interpret the formation of what they call multi-viral bodies (MViBs), avoiding any direct claims that these are "multivesicular bodies" (MVBs). Overall, their data support a "bulk release" model wherein large vesiclular structures containing many virions fuse with the plasma membrane to release many virions at once into the extracellular space.

The imaging methods are cutting edge and provide a new level of visual detail by which herpesvirus researchers can appreciate the dynamic process of HCMV egress from fibroblasts. That said, a weakness of the paper is that not much is really done to perturb any of the key cell biological processes in order to test a specific hypothesis for how so called MViBs are formed and how egress works.

We thank reviewer #3 for the supportive perspective on our manuscript. The reviewer raises a similar concern as reviewer #2. As stated above, we tested a range of inhibitors in an effort to identify the molecular pathway involved in MViB morphogenesis in response to the first round of reviews. However, as we describe in the manuscript, with limited success (Sup. Fig. 9, lines 318-334 and 483-495).

On a related note, the work observes the process in a single cell type-- fibroblasts, with a single HCMV strain TB40/E. Although it is remarkable that bulk release may well account for the predominate mode of HCMV virion egress from the infected cell, this observation appears to be the core central finding of the paper and no efforts are made here to determine in a comparative way how representative the cell biology of egress studied here might or might not apply more generally to naturally circulating HCMV strains, nor even to address other laboratory adapted isolates.

We replicated the core experiments with a BAC-derived Merlin with restored UL128 and RL13 expression. The results (Fig. 6, Sup. Fig. 10-11, lines 336-345) and conclusions (lines 383-390, 403-411) are included in the revised manuscript and indicate that Merlin produces similar or even higher amounts of MViBs but fewer EVAs as outlined in response to reviewer #2.

Also, when the authors write they do not know if the MViBs "might be a degradation pathway," seems like a bit of a forced "straw man" argument used to frame their work as hypothesis based, when in fact some of the outcomes were likely obvious to begin with.

Reviewer #3 raises an interesting concern. We disagree, however, that the assumption of a large, multivesicular structure being targeted for degradation is generally unreasonable, as it was recently also discussed by Momtaz et al. [5]. We agree however, that MViBs are less likely to be targeted for degradation. Still, this point came up in the previous round of reviews, and we would therefore like to keep it included. We added lines 133-134 to our manuscript to clarify this.

* Please note that I was not a reviewer of the original submission of this manuscript. I am providing my initial views on the current submission.

**Part II – Major Issues: Key Experiments Required for Acceptance**

**Reviewer #1**:

I confess that I am not familiar enough with advanced imaging techniques to make technical comments. I am convinced that the structures described actually exist. Beyond my sense that these MViBs might represent a more complete characterization of the traditionally conceived “AC”, I am concerned that these studies make use of a single strain of HCMV. In several places the authors note the dramatic differences between HCMV strains in terms of envelope composition and modes of spread, and seem to offer these as important to the significance of their studies. I agree that differences in the assembly/egress compartment might go a long way to explaining strain variation. Therefore, it is disappointing that the studies involve exclusively TB40-BAC4. I understand that these are technically difficult experiments, in part because of the need for a dual fluorescence markers. I am reluctant to suggest that they must include other strains prior to publication. But a more comprehensive and accurate discussion of the literature as pertains to inter-strain variation would seem like a reasonable request. Addressing some of my “minor points” would be a good place to start.

This point has been raised above, and we have repeated the core assays with a repaired Merlin expressing pentamer and RL13. The data (Fig. 6, Sup. Fig. 10-11, lines 336-345) and discussion (lines 383-390, 403-412) can be found in the revised manuscript. Moreover, we thank reviewer #1 for his valuable suggestions and improved our introduction and discussion accordingly (lines 79-101 and 505-516).

Reviewer #2: See above

Reviewer #3: Fig 4 and Lines 213-242: the CD63-pHluorin system is well described in the text. However, the data presented do not match the narration--which leads a reader to believe that a "flash" will be observed from the CD63-pHluorin channel when an MViB fuses with the plasma membrane to release viral cargo to the extracellular space. Neither SI Figure 8 or the Figure 4 panels themselves reveal any "flash" .. and no live cell imaging is shown that supports this claim, either. To be clear, this reviewer is not skeptical of the claims or model. But I do not find that the data presented support the claim.

We apologize for not describing this figure more accurately. Fig. 4 only shows frames before and after MViB fusion. However, membrane fusion is indicated as a spike of relative CD63pHluorin fluorescence in Fig. 4C at around 100 seconds. Suppl. Vid. 8 is a rendering of a fixed 3D immunofluorescence dataset without dynamic information. The pHluorin shown in this sample is constitutively fluorescent as fixation disables the proton pumps responsible for maintaining a low pH in acidified cellular compartments. We revised the text to make these points clearer (lines 265-268 and 1271-1273). We also added Sup. Vid. 9 showing lattice light-sheet microscopy data of a fibroblast cell exocytosing MViBs while moving over the growth substrate.

It would be important to show this "bulk release" phenomena occurs in more than one cell type and for different strains. Right now all the data are from TB40/E in fibroblasts. How does this look for strains like BAC cloned repaired to fully clinical status Merlin or TR3 strains? TB40/E is likely to have adapted to cell culture in a manner that promotes high level release of cell free virions, so it is uncertain whether the data shown here are representative of "fully clinical" naturally circulating HCMV strains.

We included new data using a BAC-derived Merlin with restored UL128 and RL13 expression (Fig. 6, Sup. Fig. 10-11, lines 336-345) and discussion (lines 383-390, 403-412) as outlined above.

The authors also raise points about genes like UL135 which they argue might be important for the genesis of virus filled MVB like structures. Why not test some of these ideas? It seems unlikely for instance that AD169 would fail to generate them, even though it lacks UL135 and many other ULb' genes.. Overall the study would be more valuable if the authors showed or at least tested if some viral genes might be important for driving bulk release versus other egress modalities.

Bughio et al. did already show that UL135 is important for the biogenesis of virus-filled MViBs in TB40-infected endothelial cells [6]. This manuscript focuses on MViBs as part of a so-far neglected bona-fide egress pathway. We agree that it will be important to dissect the molecular determinants of this egress pathway. As outlined in the first response to reviewer #2, we tried to narrow down involved pathways using inhibitors which was unsuccessful. Therefore, there are no clear leads right now which factors determine potentially different HCMV egress pathways. Our study lays important groundwork but future studies will be needed as defining these determinants is beyond the scope of this work. We revised the text to better define the limitations of our work (lines 135-140 and 347-354) and added the missing reference (line 138).

**Part III – Minor Issues: Editorial and Data Presentation Modifications**

**Reviewer #1:**

• Lines 62-67. I am concerned that these statements concerning the trimeric and pentameric complex are simplified to the point of conveying erroneous information.

o Yes, it seems that cell-to-cell spread can be mediated by gH/gL/UL128-131 in the absence of gH/gL/gO; this applies to fibroblasts as well as epithelial/endothelial cells (see Laib Sampiao 2016) But, note that efficient infection of epithelial and endothelial cells by extracellular virus requires both gH/gL/UL128-131 and gH/gL/gO. (there are numerous examples of papers showing this, including Wille 2010, Zhou 2013, Kabanova 2016 and Stegmann 2017 JVI).

o Yes, it is clear that strains can vary drastically in the amounts of the gH/gL complexes, but the genetic correlates are less well understood than the statement here seems to suggest. Yes, UL148 and US16 seem to play roles in assembly and trafficking of gH/gL complexes, but this does not explain in the least the dramatic strain variance that we and others have noted. Murrell 2013 describes a G-T substitution in the UL128 of TB40/e BAC4 that affects mRNA splicing. They offer this as an explanation for the low gH/gL/UL128-131 in TB40 virions compared to Merlin BAC, but as we have noted, TR BAC is also low in gH/gL/UL128-131 but is like Merlin at this nt residue. So, this is not an adequate explanation either.

o The notion that a culture of cells infected with genetically pure isolate of HCMV releases particles that vary in the abundance of gH/gL complexes is interesting and perhaps even likely, but I’m not sure there is any clear data published that demonstrates this. Yes, the Scriviano 20212 paper concluded that there was heterogeneity in the amounts of gH/gL/Ul128-131 in the virion envelope. But there were no direct data showing this, and there are other interpretations for their observations. Moreover, the flow cytometry analyses of Vlasak 2016 seem to show a good deal of regularity in the amounts of gH/gL/UL128-131 among virions within a stock. That said, Li JVI 1995 suggested virion heterogeneity as an explanation for their observations of a reversible antibody-resistance phenotype that did not involve genetic changes to the antigen.

o Overall, I caution the authors with this kind of oversimplification as it may well detract from the significance of their work.

We sincerely thank reviewer #1 for his kind suggestions. We have not discussed the glycoprotein complexes in this detail since none of our experiments directly probe the abundance of glycoproteins. However, we agree that a more precise summary of issues relating to glycoproteins and tropism is indicated. We modified the text accordingly (lines 79-101).

• Line 108; What makes this view “unbiased?” I suspect that some readers (including me) might object to the notion that any “view” can be “unbiased.” One of the deepest insights of the quantum revolution is that it is not possible to observe anything without imputing something during the act of observation.

We originally used this wording to indicate that 3D CLEM allows us to assay the complete cellular context in contrast to traditional transmission electron microscopy, which is restricted to mostly random slices. We revised the wording (line 146).

• Fig 2G. How do you know this particle is in the act of budding/envelopment, and not “back fusing” or simply pushed into the vesicle membrane? Yes, I see the membrane curving around this particle, and that would be consistent with the model of budding, but there seems to be no way to know the fate of the structure in a static image as this.

We agree with reviewer #1 that it is practically impossible to discern these two possibilities in a static TEM image using chemically fixed samples. This is equally true for most published literature using EM to illuminate the nature of HCMV envelopment [3,7]. Still, our data indicate that MViBs are produced and released at significant rates. We, therefore, argue that it is more likely to find budding than back-fusing particles. We modified the text accordingly (lines 1241-1242).

• Fig 2E/Line 179-181; To suggest that the MViBs are the predecessor/source of EVAs based on “no significant difference” in content, seems weak given the next statement, “very heterogeneous in size and content.” If the later is true it would seem that that MViBs and EVAs would be similar in content regardless of their relationship; i.e., both are heterogeneous in size and contents.

As shown in Fig. 2E, the contents and composition of MViBs and EVAs are very similar. We changed the wording in the manuscript to make this clearer (line 218).

• Line 181. Related to the above point, if some MViBs contain only a few particles and others several hundred, what is the authors’ definition of MViB? And why is this necessarily different than the “AC”?

As described above in part I, the AC is an area containing many individual vesicles and interconnected membranes. It is, therefore, not one individual vesicle. As shown in Figs. 2E-I, 6D-E, Sup. Figs.1,2,11 and Sup. Vids. 2,4, MViBs contain a mixture of virions, dense bodies, and other vesicular material, which is different from individual small vesicles that contain one virion as mostly represented in the current literature [3].

• Fig 3/Video 5. Indeed these images are neat, but I have a hard time understanding how they are interpreted as the authors’ suggest; i.e. “relaxes at it”. I admit that this might be an instance that I do not have a trained eye for such imaging techniques.

We agree that presenting complex 4D data in a manuscript is challenging. Because the term relaxing has also troubled reviewer #2, we revised the wording (lines 156, 243, 248, 282).

• Fig 3C/Vid6-7; I accept that the authors’ can distinguish various structures in the cell such as the MViBs using their CLEM approach. And I can accept that the images in Fig 3 and Vid 6-7 show the exiting of viral entities from the cell. But I don’t see how the authors’ can say that the material exiting the cell in the live images at this lower magnification is the same material they call MViBs in the higher magnification analyses. Especially since the heterogeneity of the MViBs structures seems to defy clear definition other than a vesicle containing a wide range of number and types of viral particles. Again maybe I am missing some aspect of the analyses.

The reviewer is correct in stating that there is no direct evidence that the individual MViBs found in electron microscopy would have been released at the cell surface. This is an inherent limitation of electron microscopy as it lacks the temporal dimension. For this reason, we used 3D-CLEM, which is currently the technological gold standard, to identify specific markers that correlate with the identified ultrastructures. Subsequently, we used these markers to illuminate the faith of MViBs using live-cell microscopy. Using this approach, we supplemented static EM data with spatiotemporal information. Of course, our data is of correlative nature as no live-cell EM exists to date. While our approach is cutting-edge and has never been applied to HCMV morphogenesis, we are aware of its limitations. We modified the text accordingly to emphasize these limitations (lines 390-395).

• Line 333-334. I’m not sure I understand the practicality of the authors’ distinction between HCMV “using” MVBs by “transforming” them in to MViBs, versus “generated de novo.” Given the massive effect that HCMV infection seems to have on the secretory organelles ( a point well-appreciated by the authors; lines 77-87), this distinction seems to dissolve.

We absolutely agree with reviewer #1 that this is a fuzzy distinction. However, the last round of reviews largely focused on these problems and disregarded that HCMV largely remodels the secretory system. We greatly appreciate this reviewer’s point and modified the text accordingly (lines 415-419).

• Line 363-365. This statement implies that AD169 does not produce cell-associated virus, which depending on ones’ definition of “cell-associated”, is simply not true. One can certainly get copious amounts of infectious virus by sonication of AD169-infected cells. Likewise, we showed in Shultz JVI 2020 that TB40-BAC4-infected cells contain plenty of infectious virus, yet the spread of TB40-BAC4 was predominately via a cell-free mode; sensitive to neutralizing antibodies. We did not analyze AD169 in these studies, but I think the literature is replete with examples of AD169 spreading the presence of neutralizing antibodies, indicting some kind of cell-to-cell spread mode. Moreover, I’m not sure how the cited Murrell papers would support the authors’ statements. It is true that the BAC clone Merlin is especially good at cell-to-cell spread mechanisms, but I don’t think we understand why. Murrell 2017 suggests it is due to the high levels of gH/gL/UL128-131, but Shultz 2020 shows that is it not that simple. There could be a connection between the formation and trafficking of the MViB described by the authors’ and these spread mode differences.

We agree and thank reviewer #1 again for his helpful insight. Indeed, the definitions for cell-free and cell-associated virus populations are unclear. We changed this part (lines 450-454) and expanded the introduction and discussion on differential spreading mechanisms of HCMV strains (lines 79-103, 505-521).

• Line 411. As before, I would caution against making universal statements about HCMV biology from a study like Scriviano 2012, which involved only a single strain like TB40/e. in Schultz 2020, we demonstrated dramatic differences between the BAC clones TB40/e, TR and Merlin in cell-free and cell-associated virus in fibroblasts and epithelial cells. We did not investigate endothelial cells, but it seems likely that substantial inter-strain variation exists in this cell type also.

We rewrote the passage (lines 505-521) to avoid overly simplified and universal claims.

**Reviewer #2:**

see above

**Reviewer #3:**

Lines 101-203..."the deletion of the viral protein UL135 leads to an abrogation of virus-filled MVB-like structures, and mutating UL71, a viral protein likely being involved in membrane scission". No citation is provided to support the claim about UL135.

We apologize for the missing reference. We added it to the revised manuscript in line 138.

Furthermore, UL133-138 are dispensable for replication in culture. So, how strong is this claim in the first place? Aren't MVB-like structures seen with laboratory adapted strains like AD169 which lack UL135? Sounds like a strain-specific / cell type specific defect that should be contextualized as such and interpreted more carefully. In fact, defects in MVB formation secondary to UL135 could be indirect, since UL135-null viruses have defects in viral DNA synthesis and viral gene expression.

We thank reviewer #3 for this point. We expanded the discussion of Bughio et al. [6] accordingly (line 135-140).

Line 288: "our data indicate a novel functional egress pathway for HCMV in which MViBs are targeted for secondary envelopment and subsequently exocytosed" .. this line is imprecise. Virions within MViBs ("multi-viral bodies") would be expected to already have undergone secondary envelopment.

We thank reviewer #3 for the careful reading of our manuscript and apologize for the imprecise language. We revised the passage for clarity (lines 347-354).

Line 176-177: the idea that these large vesicles containing many virions might result from a so-far unrecognized "degradation pathway" seems far fetched and it's a little tiresome to see the argument given equal weight. Seems like a straw man.

This point has already come up in part I above. Also, the word “unrecognized” refers to the egress pathway in this context. MVBs fusing with lysosomes for degradation is a well-established process (reviewed in [8]). Even though we agree with reviewer #3 that MViBs are targeted for degradation is rather unlikely, we would still like to keep this point included as it has come up in previous work [6,9], and in the first round of reviews. We modified lines 133-134 to accommodate this reviewer’s perspective.

References

1. Das S, Vasanji A, Pellett PE. Three-Dimensional Structure of the Human Cytomegalovirus Cytoplasmic Virion Assembly Complex Includes a Reoriented Secretory Apparatus▿ †. J Virol. 2007;81: 11861–11869. doi:10.1128/jvi.01077-07

2. Das S, Pellett PE. Spatial Relationships between Markers for Secretory and Endosomal Machinery in Human Cytomegalovirus-Infected Cells versus Those in Uninfected Cells. J Virol. 2011;85: 5864–5879. doi:10.1128/jvi.00155-11

3. Schauflinger M, Villinger C, Mertens T, Walther P, Einem J. Analysis of human cytomegalovirus secondary envelopment by advanced electron microscopy. Cell Microbiol. 2013;15: 305–314. doi:10.1111/cmi.12077

4. Procter DJ, Banerjee A, Nukui M, Kruse K, Gaponenko V, Murphy EA, et al. The HCMV Assembly Compartment Is a Dynamic Golgi-Derived MTOC that Controls Nuclear Rotation and Virus Spread. Dev Cell. 2018;45: 83-100.e7. doi:10.1016/j.devcel.2018.03.010

5. Momtaz S, Molina B, Mlera L, Goodrum F, Wilson JM. Cell type-specific biogenesis of novel vesicles containing viral products in human cytomegalovirus infection. Biorxiv. 2020; 2020.12.10.420711. doi:10.1101/2020.12.10.420711

6. Bughio F, Umashankar M, Wilson J, Goodrum F. Human Cytomegalovirus UL135 and UL136 Genes Are Required for Postentry Tropism in Endothelial Cells. J Virol. 2015;89: 6536–6550. doi:10.1128/jvi.00284-15

7. Schauflinger M, Fischer D, Schreiber A, Chevillotte M, Walther P, Mertens T, et al. The Tegument Protein UL71 of Human Cytomegalovirus Is Involved in Late Envelopment and Affects Multivesicular Bodies. J Virol. 2011;85: 3821–3832. doi:10.1128/jvi.01540-10

8. Piper RC, Katzmann DJ. Biogenesis and Function of Multivesicular Bodies. Cell Dev Biology. 2007;23: 519–547. doi:10.1146/annurev.cellbio.23.090506.123319

9. Momtaz S, Molina B, Mlera L, Goodrum F, Wilson JM. Cell type-specific biogenesis of novel vesicles containing viral products in human cytomegalovirus infection. J Virol. 2021. doi:10.1128/jvi.02358-20
